# Design of pH Responsive Textile as a Sensor Material for Acid Rain

**DOI:** 10.3390/polym12102251

**Published:** 2020-09-30

**Authors:** Viktor Stojkoski, Mateja Kert

**Affiliations:** Department of Textiles, Graphic Arts and Design, Faculty of Natural Sciences and Engineering, University of Ljubljana, 1000 Ljubljana, Slovenia; viktorstojkoski@icloud.com

**Keywords:** pH sensitive dyes, bromocresol green, polyamide 6, water and oil repellency, acid rain

## Abstract

The chemical composition of rainwater can serve as an indicator of the excess of acidifying air pollutants. The pH value of rainwater in the presence of sulphur dioxide and nitrogen oxides, the precursors of acid rain, falls below pH 5.6, which is the limit value for acid rain. In this research, the tailoring of halochromic textile was examined for the design of a functional textile that can serve as a sensor and inform the wearer about the presence of pollutants in the air by means of an immediate colour change. For this purpose, a polyamide 6 fabric was dyed with the pH-sensitive Bromocresol green dye, which causes a colour change below pH 3.6 (yellow) and above pH 5.4 (blue). In addition, the dyed polyamide 6 fabric was treated with a water and oil repellent finish. Colour and colour change before and after immersion of unfinished and finished dyed samples in buffer solutions with different pH values were evaluated spectrophotometrically using the CIELAB colour space. The colour fastness to rubbing, washing, and light, and the water and oil repellency of the dyed fabrics were determined according to valid SIST EN ISO standards. The results showed that the unfinished dyed polyamide 6 fabric undergoes a reversible colour change faster and more clearly than the finished dyed polyamide 6 fabric. The dyed polyamide 6 fabric had good colour fastness to rubbing and domestic and commercial laundering, while the colour fastness to light was poor. In addition, the dyed polyamide 6 fabric was pH-sensitive, despite dye degradation under xenon light, regardless of whether it was finished.

## 1. Introduction

During the second half of the previous century, gradual but major changes in rainwater and groundwater quality occurred as a result of an excess of environmentally harmful atmospheric emissions associated with rainwater acidification. After extensive and comprehensive studies to gain a substantial understanding of how acid deposition alters ecosystems, it was found that air pollution is closely related to rainwater quality. The pH of rainwater drops and becomes more acidic due to atmospheric emissions of air pollutants, almost exclusively caused by destructive human activities, in particular the burning of fossil fuels, industrial and agricultural activities, and car exhaust fumes. The phenomenon of wet deposition with high concentrations of sulphur dioxide and nitrogen oxides has been defined as acid rain [1,2,3]. Rain is categorised as acid rain when its pH is 5.6 or below. The acid rain phenomenon remains topical, despite extensive research and continuous monitoring of acid deposition and air pollution being carried out for at least half a century, following several cross-border agreements, protocols, and policies [1]. Although emissions have decreased significantly in Western European countries, North America, and some others, Southwest China and Southeast Asia countries are among the worst affected areas [2].

Smart textiles and apparel, and wearable technology, have become objects of significant interest and investment, particularly in the last six years. This is evident in both the scientific and industrial communities by the emergence of a wide range of new products [4]. Smart textiles can sense, react, or adapt to external stimuli [5]. Chromic materials are an important part of smart materials and are defined as materials that undergo reversible colour change triggered by external stimuli [6]. Chemochromic materials, so-called halochromic materials, are sensitive to changes in pH, such as pH-sensitive dyes. These dyes are sensitive to the hydrogen ion (H^+^) and are called halochromic dyes [7]. The most important classes of pH-sensitive dyes are phthalides, fluoranes, triarymethines, and simple azo dyes [8]. However, pH-sensitive chromic textiles are perspective materials because they can be used as flexible sensor systems that provide an easily visible signal. Several studies have focused on the combination of pH-sensitive dyes and textile materials [9,10,11,12,13,14,15,16,17,18,19]. pH indicators have been successfully applied to textile substrates by conventional exhaust dyeing [11,12,13,14,18] and the sol-gel method [9,12,13], or have been added to a polymer solution prior to electrospinning [10,12,13,17,18]. Cotton, polyamide 6, and polyamide 6.6 were typically used in these previous studies. Halochromic behaviour was generally observed on all textiles with deviations of varying magnitudes. It is strongly influenced by the dye structure [11,12,14] and substituents in the dye [11]. The influence of both the constructional properties of the fabric [13,14] and different dye–fibre interactions [12] on the halochromic behaviour, and the treatment process after dyeing [14,18] to avoid bleeding of pH indicators in wet processes, has been investigated in detail.

In addition to textiles, polyamides are used for a wide range of applications [20,21]. Different finishing processes can be used to apply different properties, such as waterproof, windproof, and breathable properties, to polyamide fabrics, thus polyamide is most commonly used for outdoor applications [22]. Furthermore, the global production of polyamide increased from 3.74 million tonnes in 1990 to 5.4 million tonnes in 2018, and polyamide accounted for around 5% of the worldwide market for fibre production in 2018 [23].

From the available literature, no study was found on the application of pH-indicator dye to polyamide 6 (PA6) fabric that could be used as an indicator for acid rain. The aims of this study were to select a suitable pH-indicator dye, dyeing process, and textile auxiliaries to achieve good dye uptake and levelling of PA6 fabric that could offer visible colour change within a pH range of 4–6. Moreover, functionalisation of dyed PA6 fabric to achieve water and oil repellent properties without deterioration of pH responsiveness was also studied. Consequently, the wearer of such textiles could be informed about the pH value of rainwater, which is closely related to the content of pollutants in rainwater. It was assumed that Bromocresol green (BCG) dye applied to PA6 fabric could serve as a good indicator for acid rain, due to its visible change of colour which occurs at pH below 3.6 (yellow) and above 5.4 (blue) in aqueous solution. This pH range is within the pH range of acid rain

The colour and colour change of dyed PA6 samples before and after finish were evaluated spectrophotometrically using the CIELAB colour space. The water and oil repellency, colour fastness to rubbing, and washing and lightfastness of dyed samples were determined according to the valid EN ISO standards. Furthermore, the lightfastness properties of dyed and finished samples were studied after specific time intervals to determine the impact of finish on lightfastness properties and the influence of exposure time in Xenotest apparatus on pH responsiveness. In addition, the response time and reversibility of the colour were investigated, because these parameters are important for tailoring the PA6 fabric for smart textile applications, such as umbrellas.

## 2. Materials and Methods

### 2.1. Fabric

In this research, 100% PA6 fabric with plain weave, mass of 67.02 g/m^2^, and density of 45 threads/cm in warp and 35 threads/cm in weft direction of the producer C.F. Weber (Leutersdorf, Germany) was used.

### 2.2. Dye

**Figure 1:**
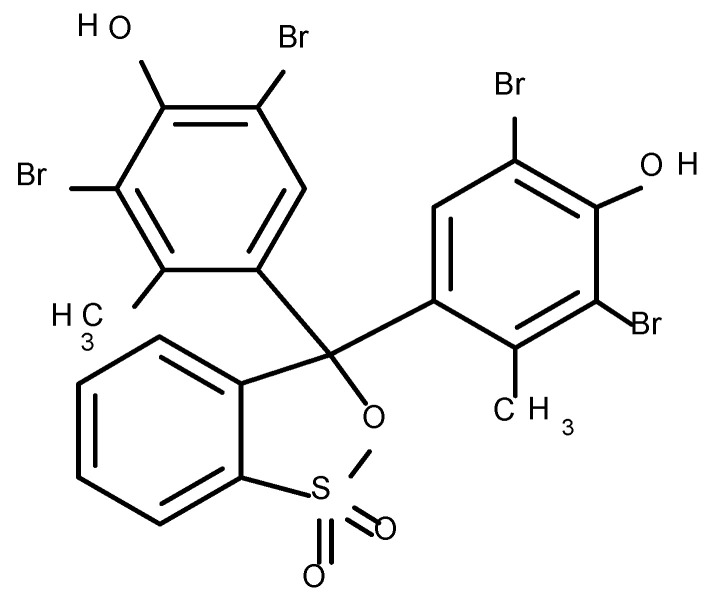
Structure of Bromocresol green.

BCG dye, as a pH-sensitive triphenylmethane dye with molecular weight of 698.01 g/mol (Honeywell Fluka, Charlotte, NC, USA), was used in the research. The molecular formula of the dye is presented in Figure 1.

### 2.3. Auxiliaries

Levelling agent Sarabid C14 (CHT, Montlingen, Switzerland), a synergistic mixture of anionic and non-ionic components, and Meropan EF 150 (CHT, Montlingen, Switzerland), an ester of carboxylic acid which ensures optimal pH condition of the dyebath during the dyeing process, were used for dyeing.

### 2.4. Finishing Agent

Water- and oil-repellency of PA6 fabric was obtained using Dynasylan F 8815 (Evonik Resource Efficiency, Bitterfeld-Wolfen, Germany), which is a fluoroalkyl-functional water-borne oligosiloxane that acts as a surface modification agent on fibre substrates.

### 2.5. Dyeing

**Figure 2:**
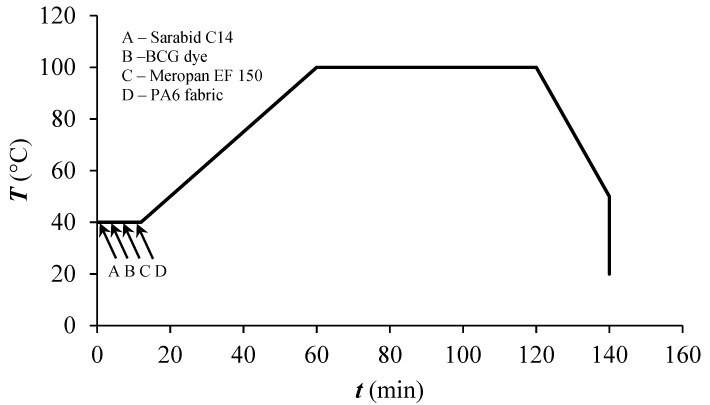
Shematic diagram of exhaust dyeing process.

Before the dyeing procedure, the PA6 fabric was washed in distilled water at 40 °C for 30 min. Dyeing of PA6 fabric mass of 5 g and 50:1 liquor-to-goods ratio was performed in a Starlet 2 (Daelim Starlet, Shiheung, Korea) apparatus using the conventional exhaust dyeing method according to the diagram shown in Figure 2.

Three different concentrations of dye, namely 0.1, 0.2, and 0.5% on mass fabric (o.m.f.) were used. Since the dye is very poorly soluble in water, 96% solution of ethanol was used for dissolving the dye prior dyeing. A quantity of 1% of levelling agent Sarabid C14 (CHT, Montlingen, Switzerland) and 0.5 mL/L of buffer system Meropan EF 150 (CHT Montlingen, Switzerland) held constant, irrespective of the dye concentration used in the dyebath. After dyeing, the dyed PA6 samples were rinsed twice in distilled water at a temperature of 25 °C. After rinsing, samples were left to dry in open air at a room temperature of 23 °C.

### 2.6. Finishing

The water and oil repellent finishing of the polyamide fabric was performed with a pad-dry-cure process. Dyed PA6 fabric was immersed in a padding bath containing 10% solution of Dynasylan F 8815 (Evonik Resource Efficiency, Bitterfeld-Wolfen, Germany) for 5 min in order to obtain good wetting. The pH value of the padding bath was adjusted to pH 4.5 with CH_3_COOH 30%. Then, padding samples were dried for 2 min at 100 °C and cured for 4 min at 150 °C.

### 2.7. Methods of Testing

#### 2.7.1. Spectrophotometric Measurements

The colour of undyed, unfinished, and finished dyed samples was analysed using a Datacolor SF 600 PLUS-CT spectrophotometer (Lawrenceville, NJ, USA). CIELAB values and reflectance (*R*) were measured whilst *K*/*S* values were calculated. All measurements were performed using four layers of fabric with a 9 mm aperture, with specular reflectance included, under D65 illumination and with a 10° standard observer. An average of ten measurements was taken for each sample. Additionally, the levelness of dyed samples was calculated by measuring *K*/*S* values of 20 random points on dyed fabric at the maximum absorption wavelength of the dye on dyed fabric (*λ*_max_ = 630 nm) according to Equations (1) and (2) [24]:

(1) $$\sigma (\lambda )=\sqrt{\frac{\sum_{i=1}^{n}{\left[{\left(K/S\right)}_{i,\lambda }-({\bar{K/S}}_{\lambda })\right]}^{2}}{n-1}}$$

(2) $$(\bar{{K/S)}_{\lambda }}=\frac{1}{n}\sum_{i=1}^{n}{\left(K/S\right)}_{i,\lambda }$$

where *σ*(*λ*) is the standard deviation of each *K*/*S* value of random points with (*K*/*S*)_*λ*_, *λ* is the wavelength of dye maximum absorption, *n* is the number of random points tested, and *(K/S)_i_,_λ_* is the *K*/*S* value of each random point. The levelness increased with the decrease of the *σ*(*λ*) value.

#### 2.7.2. Colour Fastness to Rubbing

The testing of the colour fastness of both unfinished and finished dyed samples to rubbing was performed on a Crockmeter M23888 (SDL ATLAS, Rock Hill, SC, USA) using the SIST EN ISO 105-X12:2002 standard. Colour fastness to rubbing was visually assessed using the grey scale.

#### 2.7.3. Colour Fastness to Domestic and Commercial Laundering

The testing of the colour fastness of both unfinished and finished dyed samples to domestic and commercial laundering was performed in a GyroWash apparatus (James Heal, Halifax, UK) using the SIST EN ISO 105-C06:2012 standard. Two different test methods, A1S and A1M, were used. European Colourfastness Establishment (ECE) detergent without a fluorescent whitening agent was used as a washing agent. According to the standard, the results of one multiple (M) test may in some cases be approximated by the results of up to five domestic or commercial laundering cycles at a temperature not exceeding 70 °C. Thus, the A1M method was preformed twice, corresponding to 10 domestic washing cycles. Colour fastness to washing was visually assessed using a grey scale according to SIST EN 20105-A02: 1996 and SIST EN 20105-A03:1996 standards.

#### 2.7.4. Colour Fastness to Light

The testing of the colour fastness of both untreated and treated dyed samples to xenon light was performed in a Xenotest alpha apparatus (Atlas, Rancho Cucamonga, CA, USA). Samples were prepared according to the SIST EN ISO 105-B02:2014 standard and subjected to testing.

Due to very weak fastness properties of the studied BCG dye to light, both unfinished and finished dyed samples were exposed to xenon light for 2, 4, 6, and 8 h in the Xenotest apparatus to study if the degradation of dye could influence the pH responsiveness of the dye. Therefore, the spectrophotometric measurements were performed before and after immersion of samples into buffer solutions of different pH values.

#### 2.7.5. Determination of pH Responsiveness of Unfinished and Finished Dyed Fabric

To determine the pH responsiveness of unfinished and finished dyed fabric by change of colour, which was visually and spectrophotometrically detected, the unfinished dyed samples with dye concentrations of 0.1%, 0.2%, and 0.5% o.m.f., and the finished dyed sample, dyed with a dye concentration of 0.2% o.m.f., were immersed for 1 h in buffer solutions of different pH values ranging from pH 3 to 11 (pH 3, 4, 5, 6, 7, 9, and 11). After immersion, the samples were dried for 1 h at room temperature (23 °C) and spectrophotometric measurements were then taken.

#### 2.7.6. Determination of Response Time

The response time of unfinished and finished dyed samples was recorded after immersion of dry samples into buffer solutions until the change of colour appeared. The time of reverse colour change was determined only for unfinished and finished dyed samples, which showed colour change in the expected range of pH 3 and 4. For those samples the time of reverse colour change was determined after immersion of dry samples into buffer solution of pH 7.

Additionally, the response time and spectral shifts of BCG dye in buffer solutions of different pH values was also determined. The absorption spectra of BCG in buffer solutions were recorded using UV-vis spectrophotometer Cary 1E (Varian, Australia), using a 1 cm quartz cell. Spectra were taken in the wavelength range from 400 to 700 nm.

#### 2.7.7. Water and Oil Repellency

Concerning the surface wetting of the unfinished and finished dyed samples, the SIST EN ISO 4920:2012 standard was used to determine the water repellency. To examine the oil repellency of the unfinished and finished dyed samples, the SIST EN ISO 14419:2010 standard was used. The result of the oil repellency test was the highest number of a liquid hydrocarbon at which the wetting of tested fabric had not occurred within 30 s.

## 3. Results and Discussion

### 3.1. Spectrophotometric Measurements

From the CIELAB values, gathered in Table 1, the PA6 fabric become darker, greener, and bluer after dyeing. As expected, the dye concentration influenced the CIE lightness (L*) of the dyed samples, meaning the greater the concentration, the lower the values of L*. The samples dyed with dye concentration of 0.1% o.m.f. were the lightest and the samples dyed with the highest concentration of dye (0.5% o.m.f.) were the darkest. With the increase of the dye concentration, samples became less green (values of CIE a* increase with the increase of dye concentration). The dye concentration did not significantly influence the values of CIE b*. The values of CIE b* decreased as the dye concentration increased from 0.1% to 0.2% o.m.f., meaning that the sample became bluer. At a dye concentration of 0.5% o.m.f., the sample became even less blue than the sample dyed with a dye concentration of 0.1% o.m.f., which was in contrast with our expectations. Finishing of the dyed sample with a water- and oil-repellent finish did not essentially change the colour of the sample. The dyed sample became slightly darker (CIE L* decrease), and the difference in CIE a* and CIE b* values of unfinished and finished dyed samples were in the area of experimental error.

**Figure 3:**
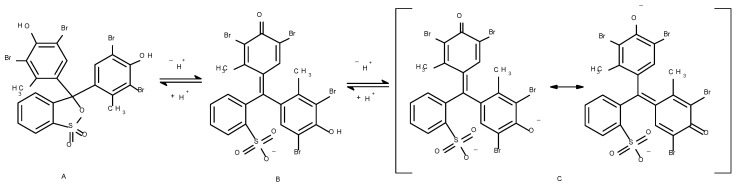
General colour changing mechanism of Bromocresol green. The neutral structure (**A**) is observed in very acidic solutions or in powder, the relevant colour change originates from a deprotonation from the single anionic form (**B**) to a double anion (**C**).

After immersion of dyed samples in different buffer solutions, the CIELAB values drastically changed at pH values of 3 and 4, while at pH values ranging from pH 5 to pH 11 only negligible changes in CIELAB values were noticed. This is expected because at pH 3 and 4 the change of colour occurs due to protonation of BCG. The dye transfers into monoanionic form (structure B in Figure 3) and the colour of the dye is yellow. Moreover, at higher pH (higher than pH 5) the dianionic form of dye (structure C, Figure 3) appears which is stabilised with resonance. The colour of the dye is blue. The mechanism is presented in Figure 3 [11].

It can be seen from Table 1 that at pH 3 all of the samples, regardless of the dye concentration used, became drastically lighter (values of CIE L* are higher), less green (values of CIE a* increased and became positive), and more yellow (values of CIE b* increased and became positive). For the PA_DF sample, only a slight increase in the value of CIE L* was noticed. The sample became greener and less blue (both CIE values a* and b* were negative), which indicates that the finishing agent influences the pH responsiveness of the studied BCG dye on PA6 fabric. At pH 4 dyed samples became darker, greener, and less yellow as evident in the decrease of all CIELAB values, compared to samples before immersion into buffer solution. At pH 4 the PA_DF sample became lighter, greener, and less blue. At pH 5 the decrease of CIE L* was noticed for all dyed samples, irrespective of the dye concentration used in the research. Values of CIE a* were negative and increased, and values of CIE b* were negative and decreased. This means that at pH 5 samples became darker, less green, and bluer in contrast to samples at pH 4. Across the range between pH 6 to pH 11, as evident from the CIELAB values, the unfinished and finished dyed samples did not exhibit any notable colour change, compared to CIELAB values of dyed samples before immersion into buffer solution. The latter suggests that at a pH higher than 5 no change of colour occurred. Unfinished and finished dyed samples were green blue.

**Figure 4:**
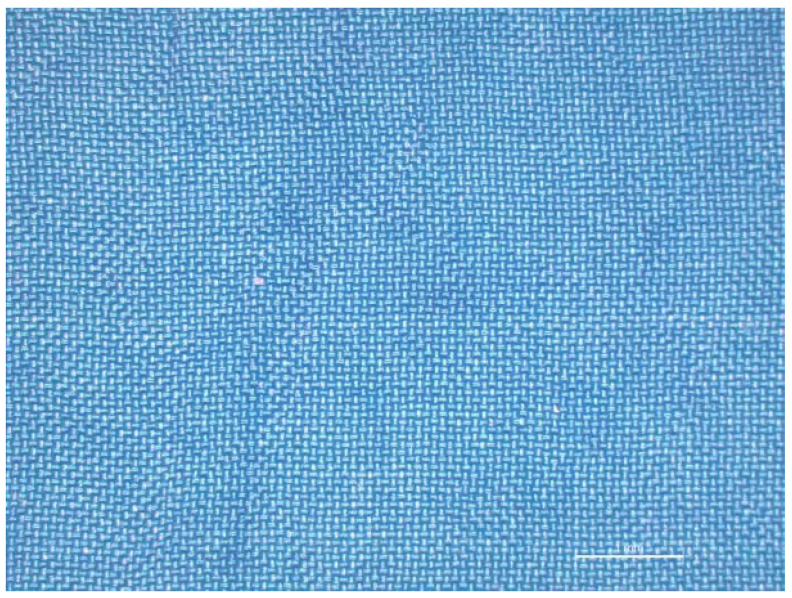
Creases on polyamide 6 (PA6) fabric dyed with dye concentration of 0.2% on mass fabric (o.m.f.).

The levelness of dyeing was determined only for samples dyed with a dye concentration of 0.2%, where 20 random measurements of *K*/*S* values were taken, and the standard deviation (*s*) was determined. The *K*/*S* value was 10.93 and *s* was 0.65. From the obtained values it can be seen that the sample is not equally dyed. The reason for higher *s* values could lie in the creases that appeared during the dyeing process on the PA6 fabric. This can be noted in Figure 4.

**Figure 5:**
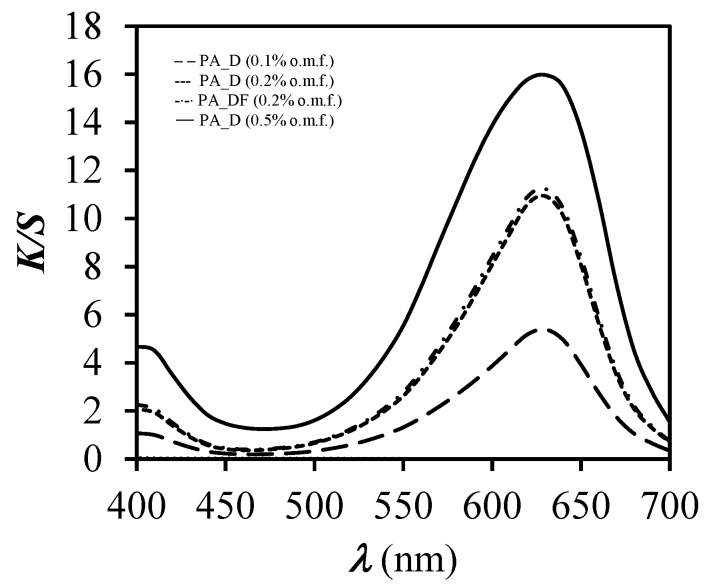
*K*/*S* values (b*) vs. wavelength (*λ*) for unfinished (PA_D) and finished (PA_DF) dyed samples of different dye concentrations

Curves of *K*/*S* vs. *λ* in the studied wavelength range from 400 to 700 nm are presented in Figure 5, which shows that the colour strength (*K*/*S*) of dyed samples increased with the increase of the dye concentration.

**Figure 6:**
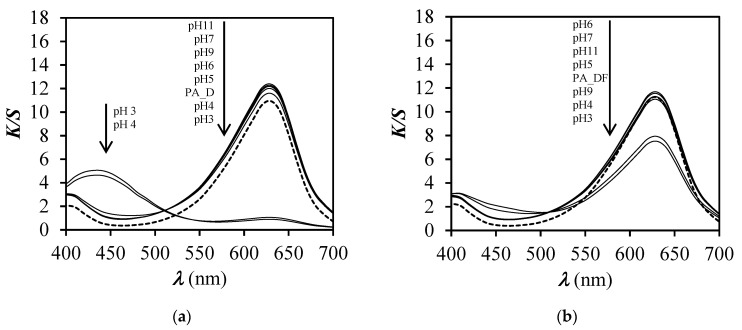
*K*/*S* values vs. wavelength (*λ*) for unfinished (**a**) and finished (**b**) samples, dyed with dye concentration of 0.2% o.m.f., before (dotted line) and after immersion (solid line) in buffer solutions of different pH values.

**Figure 7:**
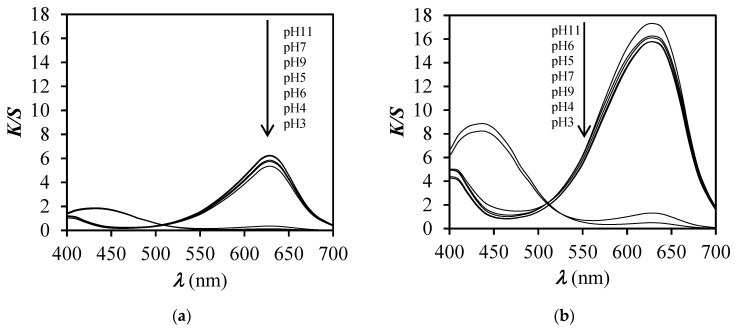
*K*/*S* values vs. wavelength (*λ*) for unfinished samples, dyed with dye concentration of 0.1% (**a**) and 0.5% o.m.f. (**b**), after immersion in buffer solutions of different pH values.

After immersion of dyed samples in buffer solutions of pH 3 and 4, the values of *K*/*S* decreased (Figure 6 and Figure 7). However, a greater decrease of *K*/*S* values was obtained for unfinished dyed samples in comparison to the finished dyed samples. These results suggest that the finishing influenced the ability of the PA6 fabric to respond to the change of pH. At pH 5 and above, values of *K*/*S* did not exhibit significant differences, meaning that the values of *K*/*S* of dyed samples were almost the same as before immersion in buffer solutions of pH 6, 7, 9, and 11.

**Figure 8:**
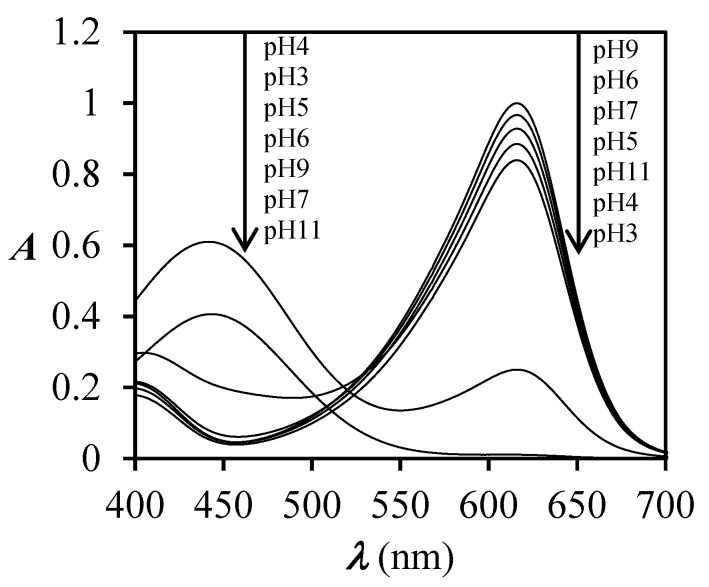
Absorption spectra of BCG dye in buffer solutions with different pH values ranging from pH 3 to 11 (pH 3, 4, 5, 6, 7, 9, and 11).

The absorption spectra of BCG on PA6 fabric (Figure 6a) expressed as *K*/*S* values vs. *λ*, showed a significant hypsochromic shift of *λ*_max_ from 630 to 440 nm when the fabric was immersed in buffer solutions of pH 3 and 4, while at other pH values no changes of spectra were noticed comparing to the dyed sample before immersion. For those spectra (pH 5–11) only a slight hyperchromic effect was noticed along the whole studied range of wavelengths in comparison with the dyed sample before immersion. In contrast to the unfinished dyed sample, the finished dyed sample showed only a hypochromic effect of *λ*_max_ at 680 nm after immersion in buffer solutions of pH 3 and 4 (Figure 6b), meaning that the sample is less pH responsive and the change of colour is less obvious compared to the case of the unfinished dyed sample (Figure 6a). With the increase of dye concentration on PA6 fabric (Figure 6a and Figure 7a,b) the hyperchromic effect of *λ*_max_ at 680 nm was noticed, which was expected due to higher adsorption of BCG dye. Moreover, at pH 3 and 4 a significant hypochromic effect occurred at *λ*_max_ = 680 nm and, at the same time, the appearance of a new absorption peak at 440 nm was noticed. This suggests halochromic behaviour of BCG on PA6 fabric and change of colour from blue to yellow-green at pH 3 and 4. With the increase of dye concentration on PA6 fabric, the hyperchromic effect of *λ*_max_ at 440 nm was more clearly noticed at both pH values 3 and 4 (Figure 6a and Figure 7a,b), which was expected. Comparison of the absorption spectra of BCG in buffer solutions (Figure 8) with absorption spectra of BCG on PA6 fabric (Figure 6a), immersed in buffered solutions of different pH values, expressed as *K*/*S* vs. *λ*, suggests that halochromic behaviour of BCG in buffer solution is slightly different to that of BCG applied to PA6 fabric and then immersed into buffer solutions. Furthermore, it should be stressed that a bathochromic shift of the absorption maximum of BCG was noticed from 618 nm (in solution) to 630 nm (in fibre) at pH 3. Moreover, the absorption spectrum of BCG in buffer solution of pH 3 showed only one absorption maximum at 445 nm, compared to BCG in buffer solution of pH 4, in which two absorption peaks appeared: The first at 445 nm and the second at 618 nm. This suggests that BCG became yellow in buffer solution of pH 3 and yellow-blue in buffer solution of pH 4. In contrast to BCG in buffer solution, the BCG on PA6 fabric immersed in buffers of pH 3 and 4 showed halochromic behaviour but to a lesser extent, because at both pH 3 and 4 two absorption peaks were noticed at 430 and 630 nm. The fabric changed colour from blue to yellow-blue. This suggests that the transformation of BCG bonded on PA 6 fabric into monoanionic form (yellow) was not as complete at pH 3 as in the case of BCG in buffer solution of pH 3, where only an absorption maximum at 445 was noticed. Moreover, the BCG showed a smaller halochromic behaviour when it was on fibre then when it was in solution. When BCG is yellow then it is in single anionic form B (see Figure 3), but when it is yellow-blue it indicates that the transformation of BCG from double anionic form C (blue), due to protonation, into single anionic form B (yellow) is not complete. The cause of this could be ascribed to both the textile matrix and the strength of dye–fibre interactions which hinder the interaction between dye and H^+^ from buffer, and reflected in lower pH responsiveness of dyed PA6 fabric.

### 3.2. Colour Fastness to Rubbing

From the results, presented in Table 2, it can be seen that both unfinished and finished dyed samples exhibited excellent colour fastness properties to rubbing. There was no staining visually detected on the dry or the wet cotton fabrics.

### 3.3. Colour Fastness to Domestic and Commercial Laundering

The results, presented in Table 3, showed that the finished dyed PA6 samples exhibited excellent (grade 5) to good (grade 4/5) colour fastness to washing comparing to unfinished dyed samples, which exhibited good (grade 4/5) colour fastness to washing, irrespective of the washing cycles used. Staining was not evident on the cotton (grade 5 to 4/5), and very little on the PA6 fabric (grade 4/5 to 4). According to the obtained results it can be concluded that the unfinished and finished dyed PA6 fabrics had very good to excellent colour fastness to domestic and commercial laundering. The number of washing cycles (w) influenced the colour fastness to washing only for the finished dyed sample, as seen in a decrease of fastness for half the grade.

### 3.4. Colour Fastness to Light

The results of colour fastness to light visually assessed using blue scale were grade 1 for both PA_D and PA_DF samples, indicated that the studied dye had very poor fastness to light and thus cannot be used for textile products which could be exposed to daily light during use for extended periods. Additionally, the fading of dyed samples was measured after 2, 4, 6, and 8 h of illumination in a Xenotest apparatus, because complete degradation of dye was noticed after 10 h. The latter was also visually detected by the change of colour of dyed fabric from blue-green to almost white.

**Figure 9:**
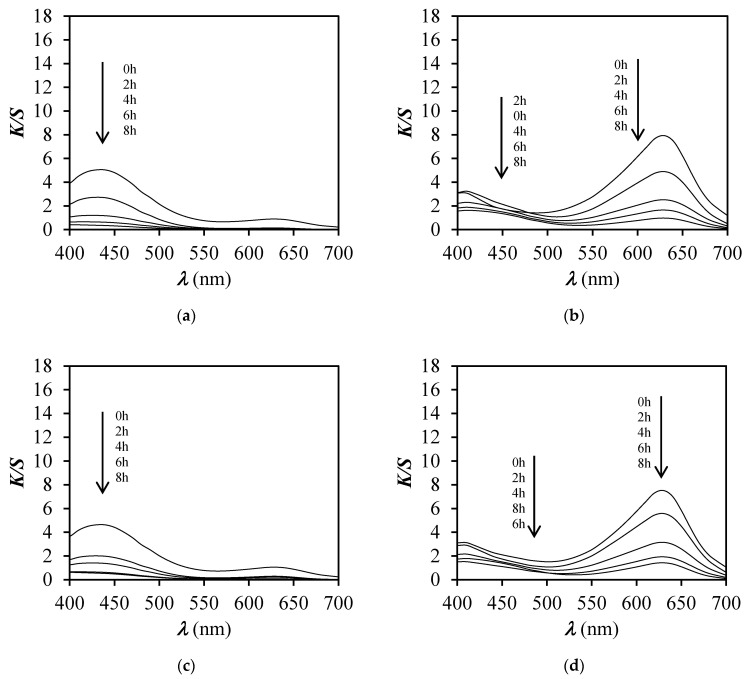
*K*/*S* values vs. wavelength (*λ*) for PA_D and PA_DF samples before and after exposure to a Xenotest apparatus for 2, 4, 6, and 8 h and immersion in buffer solutions of pH 3 and 4. (**a**) Sample PA_D at pH 3; (**b**) Sample PA_ DF at pH 3; (**c**) Sample PA_D at pH 4; (**d**) Sample PA_DF at pH 4.

**Figure 10:**
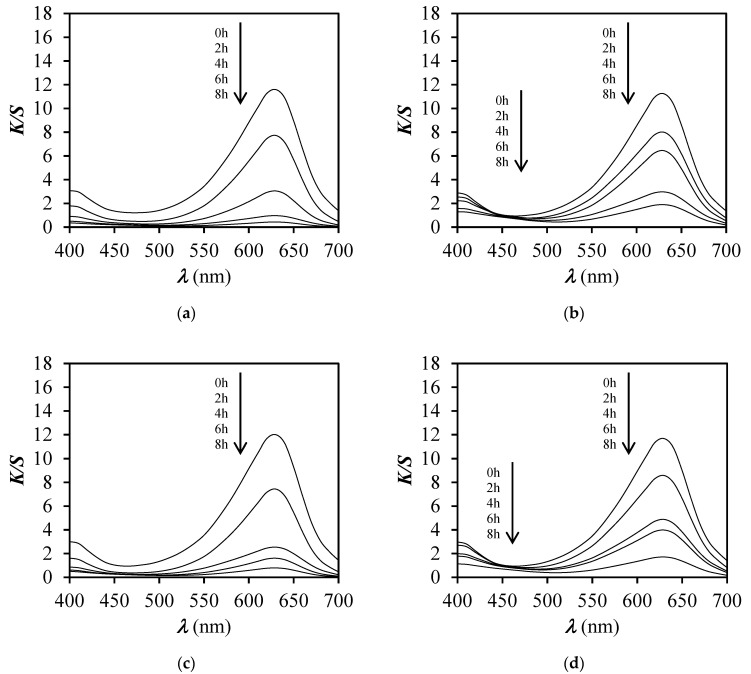
*K*/*S* values vs. wavelength (*λ*) for PA_D and PA_DF before and after exposure to a Xenotest apparatus for 2, 4, 6, and 8 h and immersion in buffer solutions of pH 5 and 6. (**a**) Sample PA_D at pH 5; (**b**) Sample PA_ DF at pH 5 (**c**) Sample PA_D at pH 6 (**d**) Sample PA _DF at pH 6.

To prove that the faded dyed sample was still pH sensitive, the illuminated dyed samples were immersed in buffer solutions of different pH values (pH 3, 4, 5, and 6) and the change of colour was spectrophotometrically determined (Figure 9 and Figure 10) after drying of samples.

The curves of *K*/*S* vs. *λ* of unexposed and exposed unfinished and finished dyed samples for a specific time to xenon light, presented in Figure 9 and Figure 10, showed that the *K*/*S* values decreased by the increase of exposure time. Nonetheless, the duration of the exposure time, whether it was 2, 4, 6, or 8 h, did not influence the pH responsiveness of dyed samples, even though dye degradation occurred. By degradation of BCG dye, the pH sensitivity was less perceived after 8 h then after 2 h of exposure, as expected.

On average, the UV index in the city of Ljubljana is 8 in June and July, 7 in May and August, 5 in April and September, 3 in March and October, 2 in February, and 1 in November, December and January [25]. Due to the poor light fastness of BCG dye on PA6 fabric, the treated fabric could only be used in winter when the UV index is lowest and air pollution is highest. In addition, the treated dyed fabric could be used as fabric for umbrellas where the light fastness properties are not as important as for functional fabrics. At this point it should be stressed that in cities such as Ljubljana the usage time for umbrellas is at least 30 min per rainy day. According to the available data, the highest number of rainy days in Ljubljana in 2019 was achieved in April, May and November [26]. Since the fabric is designed to respond to acid rain with a change of colour, the use of such an umbrella is more appropriate in winter, when the air pollution is higher than in the rainy spring season, when air pollution is lower. Such a conscious decision could prolong the lifetime of the umbrella, as the UV index in winter is 1, and the dye would therefore not degrade as quickly.

### 3.5. Response Time and Reversibility Results

The unfinished and finished dyed PA6 samples changed colour after being immersed in buffer solutions of different pH values. The colour change did not occur immediately as in the case of BCG dye in buffer solution. The colour change of polyamide 6 was gradual, and the fabric required more time for the colour change to be visually noticeable. The possible explanation for such behaviour of the BCG dye on PA6 fabric could be ascribed to both the strength of dye–fibre interaction which influences the interaction between BCG dye and H^+^ in buffer solution and the slower wetting process of the fabric with buffer solution. The same observation was noted by De. Clerk and Van der Schueren [14]. The finished dyed PA6 samples required even more time to undergo the colour change. On average the response time was 30 min for unfinished and 45 min for finished dyed samples.

All unfinished dyed samples, regardless of the dye concentration (0.1, 0.2, or 0.5 o.m.f.), reverted to their original colour. Furthermore, the samples that were previously immersed in buffer solution of pH 3 reverted to their initial colour much faster than the samples that were previously immersed in buffer solution of pH 4. The treated dyed samples with dye concentration of 0.2% o.m.f. needed a longer time to revert to their initial colour.

### 3.6. Water and Oil Repellency

Standard spray test ratings were ISO 1 for PA_D sample and ISO 5 for PA_DF sample. The ratings show that unfinished dyed samples had no resistance to surface wetting. As expected, the sample finished with Dynasylan F 8815 showed no wetting and no adherence of small drops of water on the sprayed surface.

Results in Table 4 show that the unfinished dyed sample has no oil repellency, whereas after finishing with Dynasylan F 8815 the sample become more oil repellent. After washing, the oil repellency of the dyed fabric decreased irrespective of the number of washing cycles.

## 4. Conclusions

PA6 fabric was successfully dyed with pH-sensitive BCG dye using a conventional exhaust dyeing process. The dyed PA6 fabric changed colour in the pH range between 3 and 5, indicating that the selected dye applied to the PA6 fabric could serve as an indicator of a change in the pH of rainwater. Levelness of the PA6 fabric dyed with BCG dye was not obtained due to the occurrence of creases during the dyeing process. The pH sensitivity of the dyed PA6 fabric was not affected by the dye concentration.

The PA6 fabric was also successfully treated with a water- and oil-repellent finish. The unfinished and finished PA6 showed good colour fastness to rubbing and domestic and commercial laundering. The lightfastness properties of the BCG dye applied to the PA6 fabric were not particularly good, as the dye degraded under the influence of xenon light. Nonetheless, the treatment extended the response time for the change of colour. However, the exposure time did not affect the pH sensitivity of the unfinished and finished dyed samples.

The visible signal in the case of the finished and unfinished dyed PA6 fabric can provide an alert to the presence of excessive air pollutants, thus raising awareness of the quality of the ambient air, which is crucial for human and global well-being and health.

## Figures and Tables

**Table 1:** The CIELAB colour values of undyed (PA), unfinished dyed (PA_D), and finished dyed (PA_DF) samples with different dye concentrations (c_d_) before and after exposure to buffer solutions of different pH values.

Sample	c_d_ (% o.m.f.)	pH	L*	a*	b*
PA	/	/	93.73	−0.01	2.37
	0.1	/	57.26	−21.08	−29.41
		3	79.14	−0.37	44.16
		4	74.78	−8.02	37.62
		5	56.07	−22.05	−27.46
		6	57.45	−19.96	−31.54
		7	55.74	−19.69	−32.45
		9	56.16	−19.52	−32.08
		11	55.21	−19.53	−31.99
PA_D	0.2	/	48.27	−19.23	−32.76
		3	70.53	1.24	50.92
		4	69.58	−2.63	46.72
		5	47.40	−23.00	−25.53
		6	47.91	−19.65	−32.22
		7	47.77	−18.94	−33.35
		9	47.91	−18.99	−33.14
		11	47.78	−18.54	−34.16
	0.5	/	37.23	−18.35	−24.10
		3	62.98	8.74	56.89
		4	56.95	−3.90	44.81
		5	36.24	−19.08	−21.45
		6	36.53	−16.09	−26.76
		7	38.28	−15.38	−30.11
		9	38.52	−14.97	−30.70
		11	36.62	−15.28	−28.94
PA_DF	0.2	/	47.47	−19.31	−32.23
		3	49.57	−24.67	−14.51
		4	50.20	−26.90	−8.03
		5	48.82	−20.33	−31.20
		6	48.16	−19.71	−31.92
		7	48.22	−19.50	−32.03
		9	48.83	−19.95	−31.19
		11	48.91	−19.68	−32.44

**Table 2:** Visual assessment of colour fastness of tested samples to dry and wet rubbing.

Sample	Visual Assessment Using Grey Scale	
	Staining of Dry Cotton Fabric	Staining of Wet Cotton Fabric
PA_D–warp direction	5	5
PA_D–weft direction	5	5
PA_DF–warp direction	5	5
PA_DF–weft direction	5	5

**Table 3:** Visual assessment of colour fastness of tested samples to washing.

Sample	Visual Assessment Using Grey Scale		
	Change of Colour	Staining of Polyamide Fabric	Staining of Cotton Fabric
PA_D_1w	4/5	4/5	5
PA_D_10w	4/5	4/5	5
PA_DF_1w	5	4/5	5
PA_DF_10w	4/5	4	4/5

**Table 4:** Results of oil repellency test.

Sample	Test Number of Liquid Hydrocarbon *
PA_D	1
PA_DF	6
PA_T_1w	4
PA_DF_10w	4

* 1—Mineral oil, 2—Paraffin oil:*n*-hexadecane = 63:35, 3—*n*-hexadecane, 4—*n*-tetradecane, 5—*n*-dodecane, 6—*n*-decane, 7—*n*-octane, 8—*n*-heptane.
